# Radiation Therapy with Concurrent Chemotherapy for Locally Advanced Cervical Carcinoma: Outcome Analysis with Emphasis on the Impact of Treatment Duration on Outcome

**DOI:** 10.1155/2014/214351

**Published:** 2014-11-05

**Authors:** Juan Diaz, Daohai Yu, Bizhan Micaily, J. Stuart Ferriss, Enrique Hernandez

**Affiliations:** ^1^Department of Obstetrics, Gynecology and Reproductive Sciences, Temple University Hospital, Temple University School of Medicine, 3401 N. Broad Street, Philadelphia, PA 19140, USA; ^2^Department of Clinical Sciences, Temple Clinical Research Center, Temple University Hospital, Temple University School of Medicine, Philadelphia, PA 19140, USA; ^3^Department of Radiation Oncology, Temple University Hospital, Temple University School of Medicine, Philadelphia, PA 19140, USA

## Abstract

*Objective*. To assess the effectiveness and toxicity of carboplatin concurrent with pelvic external beam radiation and low-dose rate brachytherapy and to assess the impact that adherence to the treatment plan has on outcomes. *Methods*. Retrospective chart review of 56 patients treated from January 2001 to December 2010. *Results*. Median follow-up was 68 months. Optimal dose of radiation (ORT) was defined as a minimal cervical dose exceeding 70 Gy, point A dose of 80–90 Gy, and duration not exceeding 56 days. Only 50% received ORT. In multivariable analyses we only found ORT to be statistically significant predictor for progression-free survival (PFS) and overall survival (OS) (HR [95% CI] for non-ORT vs. ORT: 2.4 [1.2, 5.1], *P* = 0.014 for PFS and 2.2 [1.1, 4.6], *P* = 0.035 for OS). The 5-year PFS in patients who received ORT was better than that in patients who received non-ORT, 56% vs. 22% (95% CI: [36%, 72%] vs. [9%, 39%]). Patients who received ORT had a better 5-year OS as well (59% vs. 33%; 95% CI: [38%, 75%] vs. [16%, 51%]). *Conclusion*. Patients with locally advanced cervical cancer treated with weakly carboplatin or cisplatin, teletherapy, and low dose-dose rate brachytherapy have poorer outcomes when treatment duration is prolonged.

## 1. Introduction

A clinical alert was released in 1999 by the National Cancer Institute (NCI) based on the results of five randomized clinical trials of concurrent cisplatin-based chemotherapy and pelvic radiation, suggesting that this approach be considered for all patients with locally advanced cervical carcinoma [1–5].

Cisplatin's mechanism of action is mediated by the formation of platinum DNA adducts. Since 1999, carboplatin, an analogue of cisplatin, with a similar mechanism of action, has been used with radiation therapy in our institution for the treatment of locally advanced cervical carcinoma. Mechanisms that underlie the interaction between the drugs and radiation therapy may include inhibition of the tumor's sublethal damage repair systems and an increase in the radiosensitivity of hypoxic cells. Compliance with treatment is very important for local control and overall survival (OS) of patients with locally advanced cervical carcinoma.

Carboplatin has decreased nephrotoxicity and neurotoxicity when compared to cisplatin and is much less emetogenic [6–12]. This favorable toxicity profile when compared to cisplatin may result in better patient adherence to the treatment plan.

We sought to assess the outcome of patients with locally advanced cervical cancer treated with carboplatin concurrent with pelvic irradiation and the impact of adherence to the treatment plan.

## 2. Materials and Methods

After Institutional Review Board approval, we conducted a retrospective chart review of patients with locally advanced stage cervical carcinoma, who received pelvic radiation therapy at our hospital between January 2001 and December 2010.

Fifty-six patients with stage IIA2 to IIIB cervical carcinoma without radiographic evidence of extrapelvic disease were identified. Stage was assigned in accordance with International Federation of Gynecology and Obstetrics (FIGO) 2009 classification. The biopsy or biopsies that established the diagnosis of invasive cervical carcinoma had been reviewed at our pathology department and the information on histologic type was retrieved from their data base.

Patients had been evaluated for the absence of extrapelvic disease with computerized tomography (CT) and chest radiograph. Treatment consisting of radiation therapy with or without concurrent chemotherapy was offered to all patients.

Carboplatin at a dose of 90 mg/m^2^ or cisplatin 40 mg/m^2^ was prescribed to be given weekly during the external beam phase of the radiation therapy. The decision to prescribe either carboplatin or cisplatin was based on physician preference. Chemotherapy and radiation therapy were initiated simultaneously. Before each chemotherapy course, complete blood cell count and serum creatinine level were obtained.

The total treatment time was measured from the beginning of radiation therapy to its completion to include brachytherapy. The “optimal dose of radiation therapy” was defined as a minimal cervical dose exceeding 70 Gy, point A dose of 80–90 Gy, with the treatment duration not exceeding 56 days [13]. Radiation treatment planning was CT-based 3D conformal for the external beam phase, as well as for brachytherapy in all cases. For the initial phase of external beam the patients were treated with a full bladder, either prone or supine, depending on the position of the small bowel relative to target volumes. The treatment planning was repeated for the bladder and rectal blocks if boost to the pelvic side walls or involved lymph node was required. The bladder was empty and the patient's position was supine or prone during the boost. The entire pelvis, that is, the entire uterus, adnexa, and pelvic nodes (external iliac, hypogastric, obturator, and presacral) up to the bifurcation of the common iliac arteries and at least 4 cm beyond the distal extension of the tumor in the vagina, was treated with anterior (AP), posterior (PA), and lateral conformal fields with 10 MeV photons with daily dose of 1.8 Gy to a total dose of 45–50.4 Gy with bladder and rectal blocks at 45 Gy if the dose exceeded 45 Gy. For bladder and rectal blocks we used AP-PA conformal fields with 18 MeV photons. Additional boost to the pelvic side wall and/or involved pelvic nodes was done with multiple 3D conformal 10/18 MeV photon fields to between 55.8 and 61.2 Gy depending on the extent of involvement with appropriate dose constraints for the bladder and large and small intestine. The boost was generally done between the two brachytherapy insertions. The brachytherapy was done with low-dose rate using cesium-137 either during or immediately after the completion of external beam radiation using two insertions separated by two weeks. The Fletcher or Nori-Hillaris applicators were used to deliver 20 Gy at the rate of 80 to 100 cGy/hour to point A with each insertion. The cumulative dose from the external beam and brachytherapy to point A was 80–90 Gy, with bladder and rectal point doses below 75 Gy and 70 Gy, respectively.

Toxicity was evaluated using the Common Terminology Criteria for Adverse Events (CTCAE) version 4.0 2009.

The patients demographic, disease, and treatment characteristics were summarized using standard descriptive statistics such as frequency and percentage for categorical variables and the mean, standard deviation, median, range, and quartiles for continuous variables. The primary outcome endpoints of this study were progression-free survival (PFS) and overall survival (OS). PFS was defined as the time from the start of the radiation therapy to death or any relapse or progression, whichever occurred first. For those patients who did not have any such events as of their last follow-up, their corresponding PFS time was censored at the time of their last follow-up. Similarly, OS was defined as the time from the start of the radiation therapy to death regardless of cause. OS was also censored if the patient had not died as of their last follow-up. Both PFS and OS were analyzed using the Kaplan-Meier product-limit method and compared between different patient subgroups with the log-rank test. The PFS and OS probabilities at five years are reported, along with their 95% confidence intervals (CIs). Cox proportional hazards regression on PFS and OS was also performed to identify which variables were of prognostic or predictive values for PFS and OS, respectively.

Independent variables under analysis included patient's age, cancer stage, histologic type, use of carboplatin (yes or no), number of chemotherapy cycles, presence of pelvic adenopathy on pretreatment imaging (yes or no), optimal delivery of radiation therapy (yes or no), and duration of radiation treatment. Stage, use of carboplatin, and optimal delivery of radiation therapy were explored with multiple Cox regression analyses with adjustments for age, number of chemotherapy cycles, and duration of radiation treatment when appropriate. Hazard ratios (HR) and their CIs were calculated for variables of interest. All data analyses were performed using SAS 9.3 (Cary, NC). A *P* value of less than 0.05 was considered statistically significant.

## 3. Results

Fifty-six patients with stage II and stage III cervical carcinoma without radiographic evidence of extrapelvic disease (IIA2 = 10 (18%), IIB = 17 (30%), IIIA = 4 (7%), IIIB = 25 (45%)) were treated during the period under study. Median age was 50 years (range: 24–83 years). Five (9%) patients had adenocarcinoma and 51 (91%) had squamous cell carcinoma. The median follow-up among survivors was 68 months (*n* = 23, range: 8–110 months).

Nonsurgical therapy consisting of radiation therapy with or without chemotherapy was offered to all patients. Nine patients (16%) received no chemotherapy. Three patients did not receive chemotherapy secondary to nonadherence to the recommended treatment and two patients declined to receive chemotherapy. The patient and treating physician decided not to use concurrent chemotherapy in one patient with chronic renal failure and in 3 patients with multiple comorbidities and poor performance status. Of the 47 patients who received chemotherapy, 43 (77% of 56) received weekly carboplatin (90 mg/m^2^) and 4 (7% of 56) received weekly cisplatin (40 mg/m^2^). The median number of chemotherapy doses given was 5 (range: 1–7). Only 28 (50%) patients received optimal radiation therapy, with the majority (24/28) of them completing radiation therapy in 8 weeks (range: 6–8 weeks). Among those who did not receive optimal radiation therapy the median number of weeks to completing radiation therapy was 11 weeks (range: 4–24 weeks; 1 patient stopped treatment after 4 weeks). Only 2 patients treated with carboplatin had grade 3 and grade 4 toxicity (Table 1).

The median age of the patients who received optimal dose of radiation therapy was 4 years older than the median age of the patients who received suboptimal radiation therapy (51.5 versus 47.5 years of age). However, this was not statistically significant (*P* = 0.56).

There were 16 pelvic failures (29%); 10 of these patients did not receive optimal dose of radiation therapy (36% of all 28 nonoptimal radiation therapy patients) while only 6 out of 28 optimal radiation therapy patients (21%) had pelvic failures. There were 10 distal failures (18%), 7 of these patients did not receive optimal dose of radiation therapy (25% of all 28 nonoptimal radiation therapy patients), compared with only 3 out of 28 optimal radiation therapy patients (11%) who had distal failures. Due to small numbers, however, these differences did not reach statistical significance.

**Figure 1:**
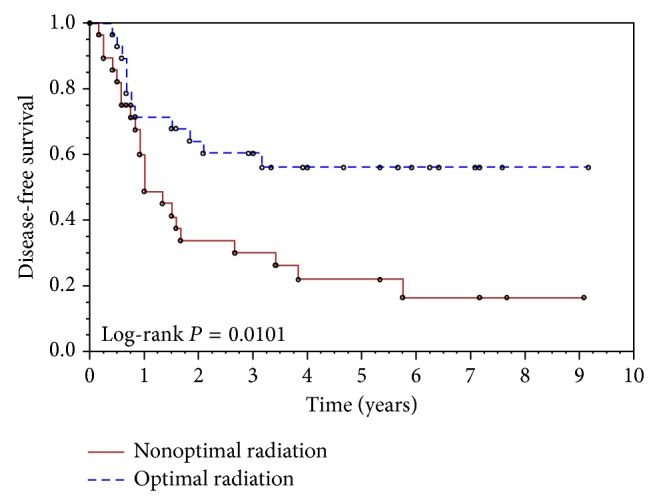
Kaplan-Meier curves of progression-free survival for optimal radiation treatment (1) versus nonoptimal radiation treatment (2).

**Figure 2:**
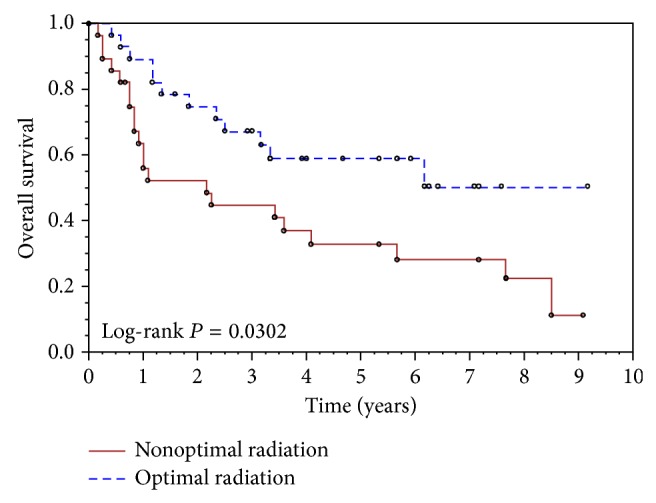
Kaplan-Meier curves of overall survival for optimal radiation therapy (1) versus nonoptimal radiation therapy (2).

Kaplan-Meier analyses of the PFS and OS showed that there was significant clinical benefit in PFS and OS for those patients who received optimal radiation therapy versus those who did not. The log-rank *P* value comparing the two radiation therapy groups reached statistical significance (*P* = 0.01 and 0.03 for PFS and OS, resp.). The 5-year PFS of the patients who received optimal radiation therapy was 56% (95% CI 36%, 72%) compared to 22% of patients who received suboptimal radiation therapy (95% CI 9%, 39%) (Figure 1). Patients who received optimal radiation therapy had a 5-year OS of 59% (95% CI 38%, 75%) compared to 33% for patients who received suboptimal radiation therapy (95% CI 16%, 51%) (Figure 2). Stage II patients had slightly better clinical outcomes than stage III patients, but these did not reach statistical significance (*P* = 0.23 for PFS, *P* = 0.39 for OS). In multivariate Cox regression analyses of stage, use of carboplatin, and optimal delivery of radiation therapy with adjustment for age, number of chemotherapy cycles, and duration of radiation treatment when appropriate only optimal radiation therapy was a significant predictor of better PFS and better OS (HR [95% CI] for nonoptimal versus optimal radiation therapy: 2.4 [1.2, 5.1], *P* = 0.014 for PFS and 2.2 [1.1, 4.6], *P* = 0.035 for OS).

## 4. Discussion

The use of cisplatin in chemoradiation regimens for the treatment of locally advanced cervical carcinoma has been the standard of care in the United States since 1999 [1–5]. Carboplatin has lower toxicity and is better tolerated than cisplatin. Carboplatin has been used in the chemoradiation treatment of other solid tumors (e.g., non-small-cell lung cancer and head and neck cancer) [14, 15].

Carboplatin has been used at our institution since 1999 for the chemoradiation treatment of locally advanced cervical carcinoma. Carboplatin has lower toxicity and is better tolerated than cisplatin. It could be administered even to patients with renal compromise, not an uncommon occurrence in patients with locally advanced cervical carcinoma. Only two of our patients who received pelvic radiation therapy with concurrent carboplatin experienced grade 3 or grade 4 toxicity.

Several phase I and phase II trials of patients with locally advanced cervical carcinoma treated with radiation therapy and concurrent carboplatin have shown minimal toxicity. Corn et al. [7] reported 15 patients treated with chemoradiation using carboplatin at a dose of 90 mg/m^2^. None of them had any grade 3 or grade 4 toxicity. We used a dose of 90 mg/m^2^ given weekly based on data accumulated prior to the routine dosing of carboplatin using the Calvert formula [16]. Dosing using the Calvert formula at an AUC of 2 as described by Higgins et al. [6] is an alternative. Higgins et al. [6] reported 31 patients with locally advanced cervical carcinoma treated with radiation therapy and concurrent carboplatin at an AUC of 2 given weekly. Only three patients developed grade 3 leukopenia, one patient developed grade 3 neutropenia, and two patients developed grade 3 thrombocytopenia. Muderspach et al. [8] reported 22 patients receiving radiation therapy and concurrent carboplatin at doses of 30, 40, and 50 mg/m^2^, twice weekly. Only 4 patients had grade 3 toxicity (two patients had anemia, one neutropenia, and one urinary toxicity). Micheletti et al. [9] reported on 12 patients with locally advanced cervical carcinoma treated with chemoradiation with carboplatin with daily doses of 12 mg/m^2^, and only two patients had grade 3 complications. Dubay et al. [10] reported 21 patients treated with radiation therapy and carboplatin at a dose of 300 mg/m^2^ every 3 weeks and only 2 patients developed grade 3 granulocytopenia, 2 patients developed grade 3 anemia, and 1 patient developed grade 3 gastrointestinal toxicity. A randomized study of patients with stage IIB-III cervical carcinoma compared radiation therapy with carboplatin alone or carboplatin with Tegafur-Uracil [11]. Weekly carboplatin at a dose of 100 mg/m^2^ was given to 231 patients. Grades 3-4 acute side effects occurred in up to 4% (1% anemia, 4% leukopenia, 2% neutropenia, and 1% gastrointestinal toxicity) of study subjects. Katanyoo et al. reported on the long-term follow-up of 148 patients with stage IIB-IVA cervical carcinoma treated with radiation and concurrent carboplatin [12]. Carboplatin was administered weekly at a dose of 100 mg/m^2^. No patient experienced grade 3 or grade 4 toxicity.

In contrast a high rate of grades 3-4 toxicity has been reported when cisplatin is used as part of the chemoradiation treatment of cervical carcinoma. Keys et al. [5] treated 183 patients with radiation therapy and concurrent cisplatin, at a dose of 40 mg/m^2^ once a week, and observed grades 3-4 toxicity (mostly hematologic and gastrointestinal) in 35% of the patients. Rose et al. [3] treated 176 patients with radiation therapy and concurrent weekly cisplatin. Forty (23%) patients had 3-4 hematologic toxicity, 12 (7%) patients had grades 3-4 gastrointestinal toxicity, and 5 (3%) patients had grades 3-4 genitourinary toxicity.

Patients with cervical carcinoma could develop subclinical changes in renal function since they are exposed to iodinated contrast media during pyelography and CT scan [17]. In addition, those with locally advanced disease may have partial or complete ureteral obstruction. An active drug with minimal nephrotoxicity is ideal for the treatment of locally advanced cervical carcinoma with chemoradiation. The more favorable toxicity profile of carboplatin when compared to cisplatin may result in better adherence to the treatment plan. However, the goal of our study was not to identify the reasons for the poor adherence to the treatment plan, but rather to evaluate the impact that adherence to the treatment plan has on the outcome of patients receiving chemoradiation for locally advanced cervical carcinoma.

In our study, patients who received optimal radiation therapy had a better probability of OS than those who did not (59% versus 33%). The difference in PFS also favored those who received optimal radiation (56% versus 22%).

The most common reason why half of our patients did not receive optimal radiation therapy was their poor adherence to the scheduled treatment visits. Several socioeconomic issues that affect our poor inner city population could explain the nonadherence of our patients to the recommended treatment plan. It has been reported that patients from low socioeconomic strata have a greater cervical carcinoma incidence and a more advanced stage at presentation, as well as lower rates of survival [18].

Amneus et al. [19] analyzed factors associated with longer treatment times on 136 patients who completed therapy. Median treatment was longer for patients who initiated brachytherapy after as opposed to during external beam radiation therapy (83 days versus 57 days, *P* < 0.0001), for patients who received interstitial versus intracavitary brachytherapy (88.5 days versus 70 days, *P* = 0.0003) and for patients who experienced an administrative delay during external radiation treatment (70 days versus 87 days, *P* = 0.005).

The median age of our patients who received optimal dose of radiation therapy was 51.5 years of age, 4 years older than the patients who received suboptimal radiation therapy (47.5 years of age). Although this finding was not statistically significant, it suggests that older women were more likely to adhere to the treatment regimen and it should be explored further. We also noted that patients with more advanced disease (11 or 38% of 29 with stage III versus 17 or 63% of 27 with stage II) were less likely to receive optimal radiation therapy.

As suggested by our study, nonadherence to the radiation therapy treatment with prolongation of the duration of treatment over 56 days is associated with poorer survival probability. Our study results are in line with the results obtained by Lanciano et al. [13] who reviewed 837 patients who received radiation therapy (without chemotherapy) for advanced cervical carcinoma. They concluded that a total dose of 85 Gy to point A delivered in less than 8 weeks improves local disease control and OS. However, some have suggested that in the era of chemoradiation and high-dose rate brachytherapy duration of treatment is no longer significantly associated with poorer outcomes [20, 21]. Shaverdian et al. [21] retrospectively analyzed the data of 372 patients with cervical carcinoma. Two hundred and six patients were treated with radiation therapy only and 166 were treated with chemoradiation. High-dose rate brachytherapy was used in 98% of patients treated with radiation only and in 85% of those treated with chemoradiation. They found that treatment duration longer than 62 days was not associated with poorer PFS or OS.

Sorbe et al. [22] reviewed 131 cases of cervical carcinoma stages I–IV. Only 36% of the patients received concurrent chemotherapy. High-dose rate brachytherapy was used. They found that total brachytherapy dose, the combined external and brachytherapy dose, and the number of the days of interruption of external radiation were all significant predictors of local tumor control. The OS of patients treated with chemoradiation was better than that of patients treated with radiation only. Song et al. [23] retrospectively reviewed the medical records of 113 patients mostly with stage II and stage III cervical carcinoma treated with concurrent chemotherapy. Most of them (95%) were treated with low-dose rate brachytherapy. On multivariate analysis, time to completion of therapy greater than 56 days was associated with increased pelvic failure (HR, 3.8; 95% CI, 1.2–16; *P* = 0.02). The 3-year pelvic failure for treatment duration greater than 56 days compared to 56 days or less was 26% versus 9% (*P* = 0.04). However, they found no association of treatment duration longer than 56 days with distal failures and disease-specific mortality. In our study, with most patients treated with chemoradiation and low-dose rate brachytherapy, we did find a statistically significant association between treatment duration longer than 56 days and survival (both PFS and OS). Most of our patients, in contrast to those analyzed on the above cited studies [20–23], received carboplatin. The 5-year OS estimate of our patients who received optimal radiation therapy (59%) is comparable to that reported by others using weekly cisplatin. For example, Rose et al. [3] reported a 4-year survival of 67% for 177 patients with stage IIB-IV cervical carcinoma without evidence of extrapelvic disease treated with weekly cisplatin and radiation therapy.

The limitations of our study are as follows: the study is a retrospective single institution study and with small sample size. In addition, patients were not randomized to optimal radiation therapy or nonoptimal radiation therapy. Therefore, our study results are empirical and exploratory in nature.

Our study of patients with locally advanced cervical carcinoma being treated with radiation (external beam and low-dose rate brachytherapy) and concurrent chemotherapy (mostly carboplatin) shows that prolongation of the interval to complete radiation therapy has a negative impact on survival. The probability of 5-year survival among those whose receive carboplatin and optimal radiation therapy is comparable to that reported for cisplatin-based chemoradiation. Although our institution and most institutions in the US now use high-dose rate brachytherapy for the treatment of this disease, low-dose rate brachytherapy is still used in many other countries. The results presented here may not be applicable when high-dose rate brachytherapy is used.

## Figures and Tables

**Table 1:** Adverse events experienced by patients treated with carboplatin (*N* = 43), according to the Common Terminology Criteria for Adverse Events (CTCAE) version 4.0. published May 28, 2009.

Toxicity grade	1	2	3	4	5
Febrile neutropenia	0	0	0	0	0
Anemia	2	6	1	1	0
Thrombocytopenia	5	0	0	0	0
Genitourinary	1	0	0	0	0
Gastrointestinal	0	0	0	0	0
